# Supplementary material for: Pre-Conceptional Anti-Thyroid Antibodies and Thyroid Function in Association with Natural Conception Rates
Source: Int J Environ Res Public Health. 2022 Oct 13;19(20):13177. doi: 10.3390/ijerph192013177 (PMC9603695; doi:10.3390/ijerph192013177)
Supplement: Supplementary file 1 [file ijerph-19-13177-s001.zip › ijerph-1965106-supplementary.pdf]

Supplementary Table S1. Distribution of serum anti-TG concentrations of the participants (n=80).

anti-TG (IU/mL)	n
<10	59
11	1
12	1
13	2
14	2
17	1
20	1
29	1
34	1
37	1
78	1
83	1
88	1
147	1
260	1
275	1
412	1
461	1
828	1
1290	1
Total	80

Anti-TG: anti-thyroglobulin antibody

Table S2. Distribution of serum anti-TPO concentrations of the participants (n=80).

anti-TPO (IU/mL)	n
<9	57
10	3
12	1
13	1
16	1
17	1
18	2
19	1
20	1
24	2
26	1
29	1
31	1
74	1
83	1
157	1
226	1
240	1
371	1
600+	1
Total	80

Anti-TPO: anti-thyroid peroxidase antibody
